# Interspecific Hybridization as a Tool to Understand Vocal Divergence: The Example of Crowing in Quail (Genus *Coturnix*)

**DOI:** 10.1371/journal.pone.0009451

**Published:** 2010-02-26

**Authors:** Sébastien Derégnaucourt

**Affiliations:** 1 Max Planck Institute for Ornithology, Seewiesen, Germany; 2 UMR CNRS 6552 Ethologie-Evolution-Ecologie, Université Rennes 1, Rennes, France; Centre de Recherches su la Cognition Animale - Centre National de la Recherche Scientifique and Université Paul Sabatier, France

## Abstract

Understanding the mechanisms that lead organisms to be separated into distinct species remains a challenge in evolutionary biology. Interspecific hybridization, which results from incomplete reproductive isolation, is a useful tool to investigate such mechanisms. In birds, interspecific hybridization is relatively frequent, despite the fact that closed species exhibit morphological and behavioural differences. Evolution of behaviour is difficult to investigate on a large timescale since it does not ‘fossilize’. Here I propose that calls of hybrid non-songbirds that develop without the influence of learning may help in understanding the gradual process that leads to vocal divergence during speciation. I recorded crows produced by the European quail (*Coturnix c. coturnix*), the domestic Japanese quail (*Coturnix c. japonica*) and their hybrids (F1, F2 and backcrosses). Most crowing patterns were intermediate to those of the parental species; some were similar to one or the other parental species, or not present in either parental species. I also observed vocal changes in hybrid crows during the breeding season and from one year to the other. This vocal variability resembles those observed during the ontogeny of the crow in quails. It is likely that similar mechanisms involved in vocal changes during ontogeny might have driven vocal divergence in the species of Palearctic quails. I suggest that hybrid crows might have resembled those produced by intermediary forms of quails during speciation.

## Introduction

During the speciation process, groups of individuals that had belonged originally to a common ancestor developed morphological, ecological and/or behavioural differences. These differences led eventually to reproductive isolation that characterizes a new species entity, according to Mayr's biological concept [1]. Two isolating mechanisms act during the speciation process [2]: postzygotic (hybrid inviability and sterility) and prezygotic (sexual selection, asynchrony of sexual cycles, habitat selection). This isolation is definitely achieved when genetic rearrangements lead to complete reproductive incompatibility. During the speciation process, when reproductive isolation is enhanced but not completed, hybridization between emerging species may occur [3]. At both morphological and behavioural levels, hybrids often exhibit a mosaic of different forms that may represent parental characteristics, intermediate characteristics as well as original forms [4]. It is interesting to point out that these morphological traits and behaviours are mainly signals assumed to be of crucial importance in sexual selection [5]–[13].

Behavioural traits that are partly socially learned might rapidly diverge between allopatric populations. Birdsong is a learned behaviour [14]–[15]. Its importance as a reproductive isolating mechanism has been well documented [15]. In songbirds, hybridization is usually seen as a result of imprinting on the song of another species [16]. Natural hybridization can lead to mixed singing, i.e. individuals singing elements of the songs of two species [17]–[19]. Cultural transmission of song characteristics in most of the oscines might have accelerated speciation [20], [21]. On the contrary, speciation processes might have been slower in species that produce vocalizations through mechanisms that are mostly under genetic control [13]. In Columbiforms and Galliforms, different experimental paradigms had apparently no effect on the normal development of the vocal repertoire [8], [22]–[25]. From these studies, it was concluded that learning had no influence on vocal development, and therefore that genetic factors are the major source of phenotypic variation [22], [26]–[28]. Such genetic determinism on call structure has also been supported by experiments of interspecific hybridization [8], [9], [28], [29].

Hybridization presents challenges to the reconstruction of phylogenies, formulation of biological species concepts and definitions [30]. Is it generally assumed that evolution of behaviour is difficult to assume on a large timescale since it does not ‘fossilize’. Nevertheless, anecdotal and scientific evidence suggest that avian vocalizations contain historical information [31]. I postulate that analysis of vocalizations produced by hybrids of vocal non-learner species may help understanding the gradual process that led to vocal divergence during speciation.

**Figure 1:**
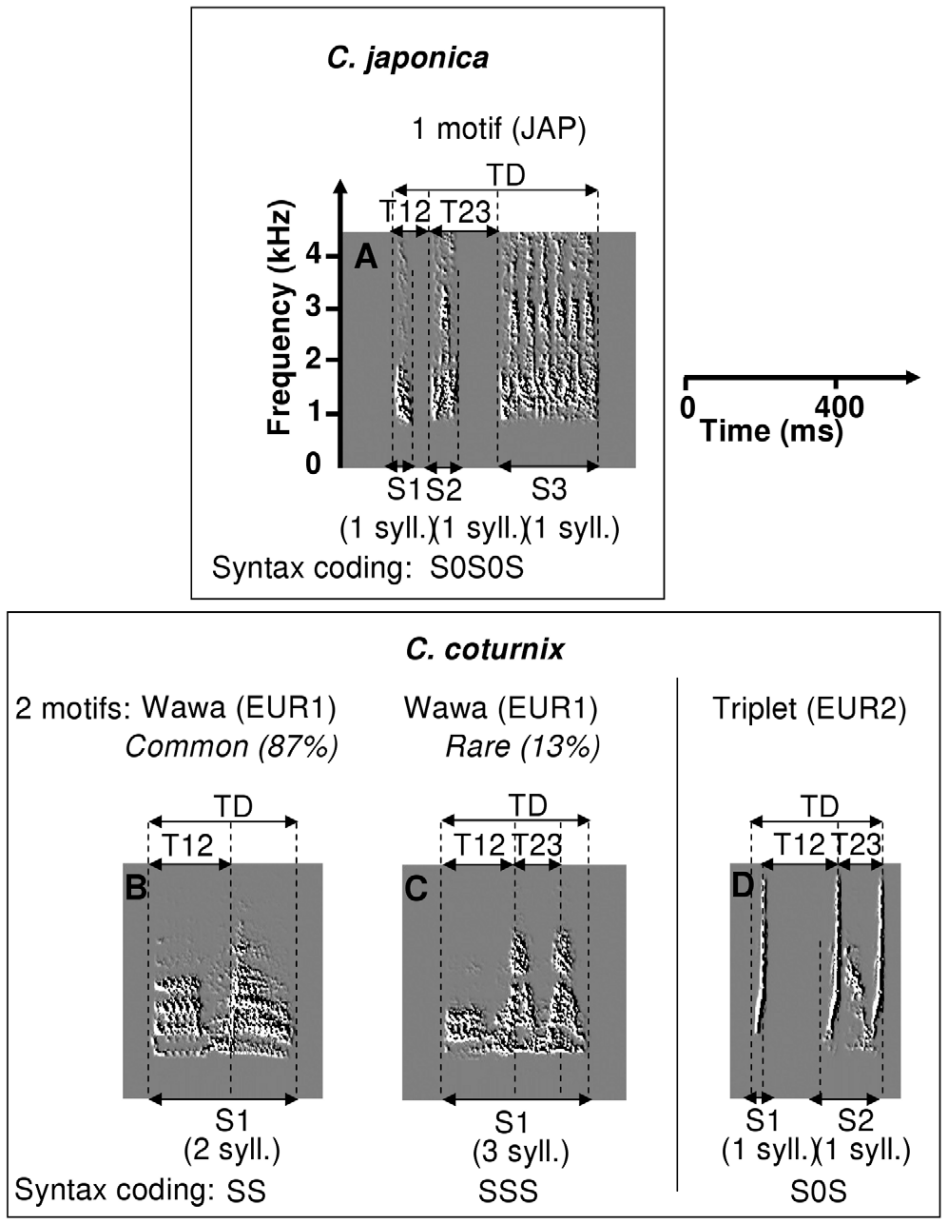
Spectrograms of crows produced by Japanese quail and European quail. A: Japanese crow; B and C: European wawa; D: European triplet. The wawa is composed of 2 (B, 87%), rarely 3 (C, 13%) syllables [39]. TD: Total Duration; S1, S2, S3: duration of Segment 1, 2, 3; T12: time interval between impulsion 1 and impulsion 2; T23: time interval between impulsion 2 and impulsion 3. Between brackets: number of syllables per segment for each motif. Below: coding of syntactical organization of the motif. S: syllable, 0: silence.

This is the purpose of this study, taking as example two subspecies of Palearctic quails: the European quail (*Coturnix c. coturnix*) and the Japanese quail (*Coturnix c. japonica*). Based on genetic analysis, we can estimate that they have probably diverged from a common ancestor between 1 and 1.5 million years ago [32]. They show a high overall similarity in morphological, behavioural and ecological features that made some authors conclude that they belong to the same species [33]. The two subspecies are allopatric, but their distributions overlap around the Lake Baikal [34], [35]. Genetic analysis on quails captured in this area confirmed that hybridization occurs between two subspecies, but details on this hybrid population (eg. degree of introgression) are still missing [36]. In the laboratory, we demonstrated that post-zygotic mechanisms have not been established yet to prevent hybridization [37]. In addition, we postulated that prezygotic mechanisms might not be strong enough to prevent hybrids pairing [38]. Both subspecies share a common vocal repertoire of different calls emitted in different socio-sexual contexts [39]. Only the structure of males' crows differ between the two subspecies. Males of the Japanese quail produce only one crow (JAP) whereas males of the European quail produce two different crows: the wawa (EUR1) and the triplet (EUR2) (Fig. 1). Divergence of vocal signals facilitating species discrimination is likely to have the largest effect on long-range signals, especially when these are used in mate attraction and territorial advertisement [40]. Influence of crow playback on female sexuality has been clearly demonstrated in quails [38], [41]–[43] enhancing its role in prezygotic reproductive isolating mechanisms. Both subspecies live in grass fields, which do not facilitate long-distance visual communication [33]. Thus, sexual partners use the acoustic channel to attract each other [38].

Quantitative analyses of acoustic features of quail crows have been done in the European quail [44] and the Japanese quail [45]. Like many birds' vocalizations used in mate attraction and territorial advertisement [46], [47], it was observed that the intra-individual variability was lower than the inter-individual variability, concluding that the quail's crow might facilitate individual recognition [44], [45]. Such individual stereotypy was also observed in crows produced by hybrid quails [48]. Hybrid crows present all intermediaries between the two crows of the European quail and the crow of the Japanese quail [28], [39], [48]. In a previous report, we used artificial neural networks to classify hybrid crows on the basis of pixel analysis of spectrograms [28]. We observed a huge inter-individual variability in crows produced by F1 and F2 hybrids. Quails issued from backcrosses produced crows similar to the European quail to which they were backcrossed, stressing again the high genetic determinism of the structure of crows [28]. This classification based on visual cues did not take into account the relevant information for acoustic discrimination by conspecifics or heterospecifics individuals, namely the spectral and temporal components of the crows. Measures on different acoustic parameters have been done for a hybrid combination: mother *japonica *× father *coturnix* [48]. Nevertheless, some methodological mistakes in this latter study (in particular, the authors did not take into account the reverberation phenomena in temporal measurements) motivated me to present new results with more hybrid combinations. In addition, computation of additional acoustic features [49] will help in describing more accurately the vocal differences between the two subspecies and their hybrids.

In several species including Quail, it has been shown that vocal characteristics may be stable from year to year, enhancing the possibility for an animal to be recognized from one reproductive season to another or even over several years [44], [50]. Nevertheless, even in non-vocal learners, some characteristics of mating or territorial calls could be modified during life [45], [51], [52]. I recorded hybrid quails at different moments of the first reproductive season and during the second breeding season. During the recording sessions, I observed significant intra-individual variability in the crows produced by some hybrid quails. It might be due to ontogenetic changes recently described in the Japanese quail [45].

In the first part, I present the vocal differences between the two subspecies and their hybrids. In the next part, I describe vocal changes in hybrid crows. The ultimate goal of this study is to use hybrid crows and recent descriptions of vocal changes during ontogeny [45], to propose a scenario for the evolution of vocal divergence of Palearctic quails during speciation.

## Results

I produced hybrids from different combinations [35]: (1) female *japonica *× male *coturni*x: H1; (2) female *coturnix *× male *japonica*: H2; (3) female H1 × male H1: F2; (4) female H1× male *coturnix*: backcross 1: BC1; (5) female *coturnix *× male H1: backcross 2: BC2. Twenty-eight male Japanese quails, 26 male European quails, 37 H1, 29 H2, 17 F2, 13 BC1 and 13 BC2 were recorded.

**Figure 2:**
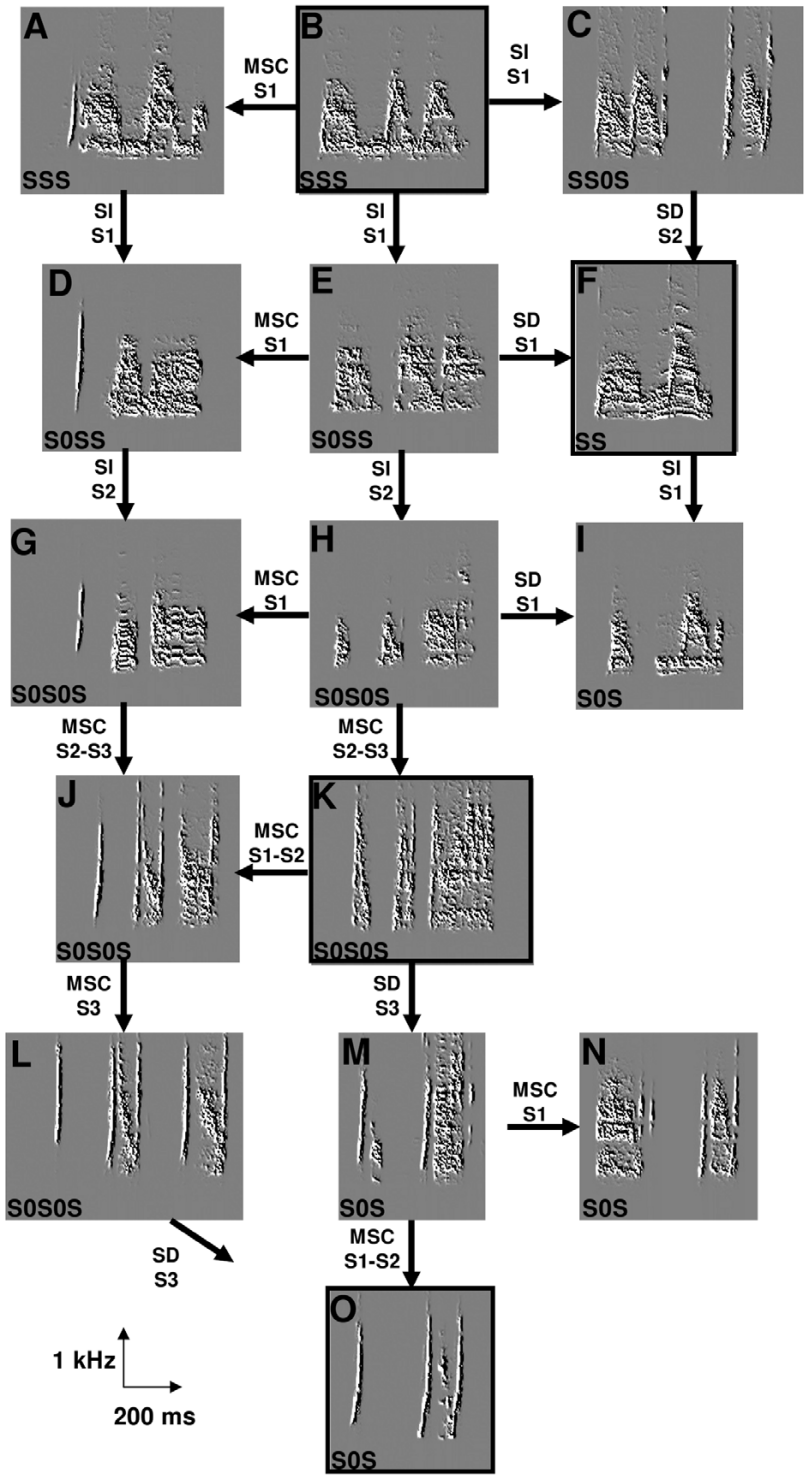
Structural organization of crows produced by hybrid quails. Hybrid crows that resemble the ones produced by the two parental species are squared. Arrows indicate transitions between the different crows. These transitions could be described using mechanisms observed during vocal ontogeny of crow [45] namely Silence Insertion (SI) and Modification of the Spectral Components (MSC). A third mechanism, Segment deletion (SD) is sometimes observed in Japanese quail (second syllable is sometimes omitted). S1 to S3: segment 1 to segment 3. Left bottom corner of each spectrogram: coding of syntactical organization of the motif. S: syllable, 0: silence. See text for details.

Figure 2 presents spectrograms of crows produced by hybrid quails of the different combinations. As previously described [28], there is a high inter-individual variability in crows produced by hybrid quails. Some hybrids produced crows similar to the crows of the European quail (Fig. 2B and Fig. 2F: wawa; Fig. 2O: triplet) or to the Japanese quail (Fig. 2K). But most of the hybrid crows exhibit all intermediaries between the three forms produced by the two subspecies (Fig. 2).

Quail crowing activity can be described hierarchically as following (Fig. 1): 1/a *syllable* is composed of different sounds; 2/sequences of *syllables* composes a *motif* (crow); 3/different renditions of *motifs* constitute a *bout*.

### Crow Bouts

**Figure 3:**
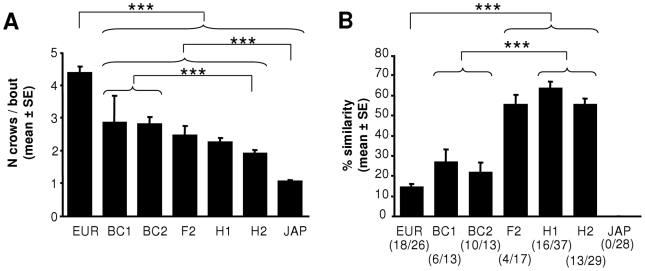
Characteristics of crowing activity in quails. A. Number of crows emitted per bout (mean±SE). B. Intra-individual similarity of acoustic structure in hybrid crows. Like European quails, some hybrid quails produce two different motifs. Number below the graph (n1/n2): n1: number of quails producing two different motifs; n2: total number of quails. ***: p<0.001. EUR: European quail; JAP: Japanese quail; H1: female *japonica *× male *coturnix*; H2: female *coturnix *× male *japonica*; F2: female H1× male H1; BC1: female H1× male *coturnix*; BC2: female *coturnix *× male H1.

The different groups differed significantly in the number of crows per bout (Fig. 3A, Kruskal-Wallis, H = 110, n = 163, p<0.001). Japanese quails often emitted a single crow per bout, rarely (and never more than) two. In contrast, European quails never produced an isolated crow: a bout was always composed of 3 to 8 crows. Hybrids exhibited intermediary values with a slight tendency for backcrosses (BC1 and BC2) to produce more crows per bout than the hybrids of the first generation (H1 and H2).

### Number of Motifs per Bird

The European quail produces two different motifs (the wawa and the triplet) whereas the Japanese quail produces only one motif (Fig. 1).

Some hybrids produced only one motif. Others like the European subspecies produced two different motifs in a same bout. There is no significant difference between the different hybrid combinations (Fig. 3B and Fig. S1, Chi-square test, chi-2 = 8.6, df = 4, p = 0.07).

### Syntactical Organization

The wawa is composed of 2 or 3 syllables, the triplet is composed of 2 syllables, and the crow of the Japanese quail is composed of 3 syllables (Fig. 1).

*Syllables* can be separated by silences or produced in the same *segment* (Fig. 1). Therefore: 1/the wawa of the European quail is composed of one segment, 2/the triplet of the European quail is composed of 2 segments, and 3/the crow of the Japanese quail is composed of 3 segments (Fig. 1).

Regarding the hybrids, the difference is significant between the groups regarding the number of motifs with 2 and 3 syllables (Table 1; Chi-square test, chi-2 = 25.4, df = 4, p<0.001). The same result is obtained if one takes into account the motifs with 2 syllables and those with more than 3 syllables (Chi-square test, chi-2 = 30.85, df = 4, p<0.001). If hybrids of the 1^st^ and 2^nd^ generations (H1, H2 and F2) produced significantly more motifs with 3 syllables (or more) than motifs with 2 syllables, the contrary is observed in backcrosses (BC1 and BC2).

**Table 1:** The different types of syntactical organization exhibited by the hybrid quails.

Crossing	S0S	SS	SSS	S0SS	SS0S	S0S0S	SSSS	SS0SS	SS0S0S	S0S0S0S	S0S0SS	S0S0SSS	2s	3s	4s	5s	total
H1	16	3	2	3	9	15	3	3	1	1	1	0	19	29	9	0	**57**
H2	9	1	2	1	9	18	2	0	1	0	0	0	10	30	3	0	**43**
F2	3	0	1	2	3	9	1	1	0	0	1	1	3	15	3	1	**22**
BC1	12	3	3	1	0	0	0	0	0	0	0	0	15	4	0	0	**19**
BC2	10	5	6	0	0	2	0	0	0	0	0	0	15	8	0	0	**23**
total	50	12	14	7	21	44	6	4	2	1	2	1	62	86	15	1	**164**

‘S0S’ indicates that the motif is composed of 2 syllables (code S) separated by a silence (code 0). 2s, 3s, 4s and 5s: total number of motifs composed respectively of 2, 3, 4 and 5 syllables. H1: female *japonica *× male *coturnix*; H2: female *coturnix *× male *japonica*; F2: female H1 × male H1; BC1: female H1 × male *coturnix*; BC2: female *coturnix *× male H1.

Regarding the syntactical organization of these motifs, those composed with 2 isolated syllables (Table 1, S0S) are more common than those with 2 syllables merged (SS). The motifs with 3 isolated syllables (S0S0S) are more common in hybrids of the 1^st^ and 2^nd^ generation.

Of the 12 different syntactical organizations observed, 11 were exhibited by the H1, 8 by the H2, 9 by the F2, 4 by the BC1 and 4 by the BC2 (Table 1).

### Specificity of Vocal Characteristics

I observed significant differences between groups for all but two acoustic features (see Material and Methods for further details; Table 2). For several parameters, hybrids showed intermediate values between the two crows of the European quail and the Japanese quail's crow (Fig. S2 and Fig. S3).

**Table 2:** Mean values (±SEM) for all acoustic variables. Kruskal Wallis results for differences between groups.

Acoustic Feature	JAP	H1	H2	F2	BC1	BC2	EUR1	EUR2	Chi2	P
**S1 (ms)**	63±18	193±17	161±8	119±1	195±40	224±36	383±24	49±38	54.1	***
	(b)	(c)	(b,c,d)	(b,d,e)	(a,b,c,d,e,f)	(a,b,c,d,e,f)	(a)			
**S2 (ms)**	59±15	182±9	168±11	175±11	182±3	173±5	-	172±17	51.5	***
	(a)	(b,e,f)	(b,c,e)	(c,d,e,f)	(a,b,c,d,e)	(a,b,c,d,e)	-	(e)		
**S3 (ms)**	328±20	182±8	180±21	204±8	-	161	-	-	74.8	***
	-	(b)	(b,c)	(b,c,d)	-	-	-	-		
**TD (ms)**	626±14	503±15	507±12	503±22	414±17	417±20	383±24	407±10	62.3	***
	(c)	(d)	(d)	(d)	(a,b,c,e)	(a,b,c,e)	(a)	(a,b)		
**T12 (ms)**	163±16	191±9	177±10	151±16	211±5	204±6	203±15	242±17	47.1	***
	(b)	(a,b,c)	(b,c)	(b)	(a,b,c,d)	(a,b,c,d)	(a)			
**T23 (ms)**	171±13	166±11	175±8	151±16	127±3	133±4	138±22	122±6	102.1	***
	(c)	(a,d)	(a,c,d,e)	(a,d,e,f)	(a,b,c,g)	(a,b,c,d,f,g)	(a)	(a,b)		
**Mean mean frequency (Hz)**	2598±57	2361±44	2608±33	2310±64	2164±69	2118±73	1731±30	2525±85	73.5	***
	(b,c)	(b,d)	(c)	(b,d,e)	(a,c,d,e,f)	(a,c,e,f)	(a)	(b)		
**Mean_entropy (deg)**	−2.6±0.1	−2.9±0.1	−3±0.1	−3.7±0.1	−3.6±0.1	−3.8±0.1	−3.9±0.2	−3.2±0.2	82.1	***
	(c)	(b,c,d)	(d)	(b,e)	(a,b,c,e)	(a,b,c,e)	(a)	(b)		
**Mean FM (deg)**	45.8±0.6	42.3±0.8	46.5±1	45.1±1.3	47.8±2	44.8±2.3	31.8±1.4	52±1.3	63.7	***
	(c)	(d)	(c,e)	(c,d,e,f)	(a,b,c,e,f,g)	(a,c,d,e,f,g)	(a,b)			
**Mean AM (×10^−4^ deg)**	59±7	57±7	57±5	54±7	55±8	53±9	56±12	48±6	101.5	***
	(b)	(a,b,c)	(a,c)	(a,d)	(a,b,d,e)	(a,b,d,e)	(a)			
**Var. mean frequency (kHz)**	642±37	598±42	628±45	468±58	577±67	688±51	611±35	606±53	23	0.3 NS
**Variance entropy (deg)**	0.67±0.04	0.66±0.04	0.76±0.04	0.9±0.15	0.96±0.08	0.98±0.09	0.41±0.03	1.21±0.07	78.4	***
	(b)	(b,c)	(b,c,d)	(b,c,e)	(a,b,e,f)	(a,d,e,f)	(a)			
**Variance FM (deg)**	577±11	614±9	589±11	586±19	565±15	580±18	537±15	563±17	8.8	0.002
	(b,c)	(d)	(b,c,d,e)	(a,b,c,d,e)	(a,b,c,e,f,g)	(a,b,c,e,f,g)	(a)	(a,b)		
**Variance AM (×10^−4^ deg)**	55±4	46±4	53±5	56±6	68±6	53±7	62±4	52±6	8.8	0.3 NS

Means with the same letter are not significantly different, Mann Whitney, p<0.05. S1, S2, S3: duration of Segment 1, 2, 3; TD: Total Duration; T12: time interval between impulsion 1 and impulsion 2; T23: time interval between impulsion 2 and impulsion 3. H1: female *japonica *× male *coturnix*; H2: female *coturnix *× male *japonica*; F2: female H1× male H1; BC1: female H1× male *coturnix*; BC2: female *coturnix *× male H1; EUR1: wawa of the European quail; EUR2: triplet of the European quail; JAP: *japonica*.

**Figure 4:**
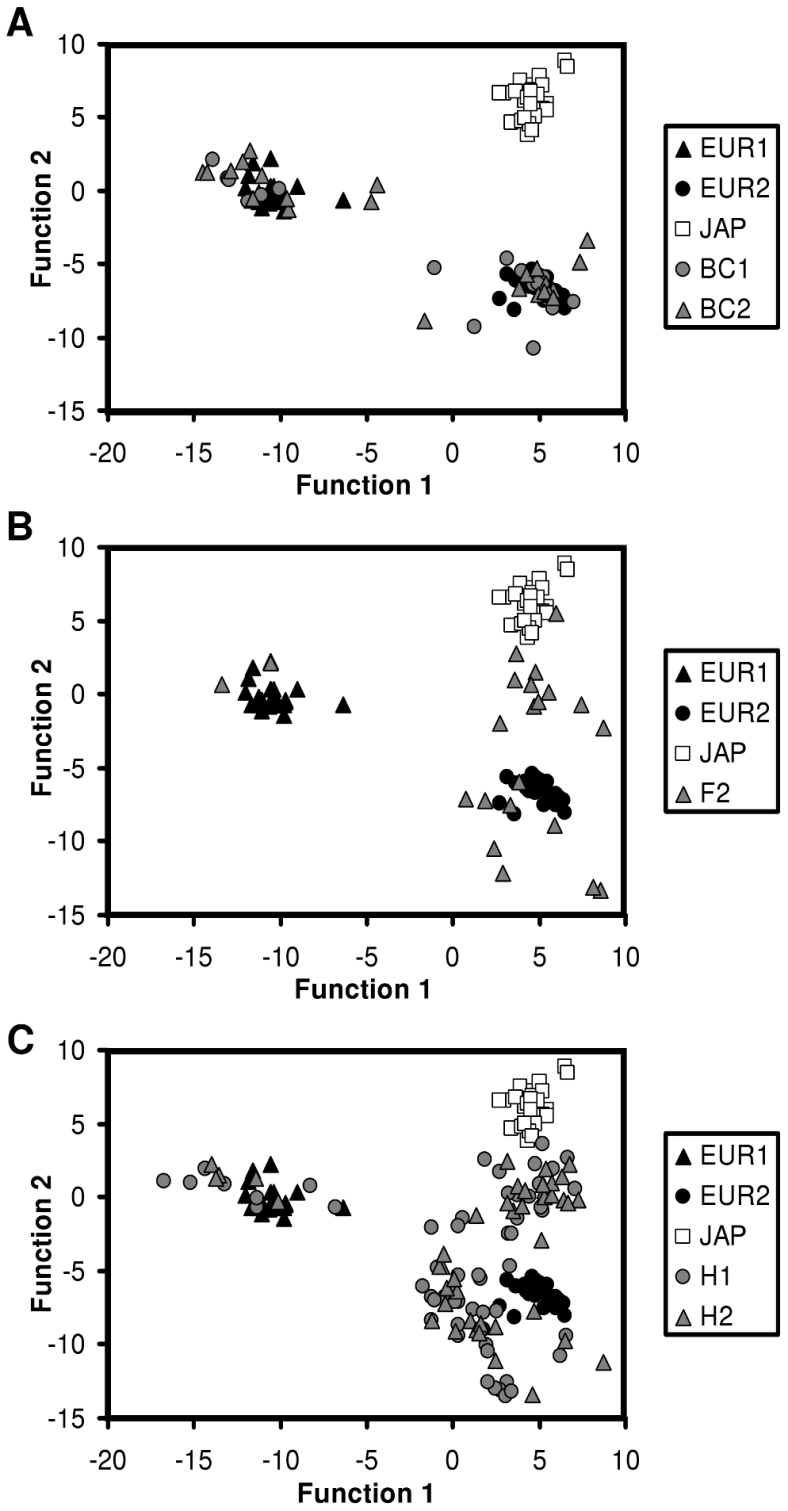
Discriminant function analyses applied to the acoustic parameters of crows. A. Calls produced by the European quail, the Japanese quail and backcrosses. B. Calls produced by the European quail, the Japanese quail and hybrids of second generation. C. Calls produced by the European quail, the Japanese quail and hybrids of first generation. H1: female *japonica *× male *coturnix*; H2: female *coturnix *× male *japonica*; F2: female H1× male H1; BC1: female H1× male *coturnix*; BC2: female *coturnix *× male H1; EUR1: wawa of the European quail; EUR2: triplet of the European quail; JAP: *japonica*.

A discriminant function analysis (DFA) was constructed on the three calls types (wawa and triplet of the European quail; Japanese crow; Fig. 4). I observed that temporal parameters contributed primarily to the two functions of the DFA (Table 3). One hundred per cent of the crows produced by the two subspecies were assigned to the correct crow type (Fig. S4). I then run the DFA without initially assigning the crows produced by the different hybrid combinations to a particular group. Crows produced by hybrids in the 1st and 2nd generations were almost equally distributed among European and Japanese quail types (Fig. S4A). No crow produced by the backcrosses (BC1 and BC2) was assigned to the Japanese type. Then, hybrid crows from all combinations were initially assigned to a separate group. European and Japanese crows were still assigned to the correct group, and crows produced by hybrids distributed among the four groups, still exhibiting differences between combinations (Fig. S4B). When the DFA was run using only spectral features, the classification slightly changed but the same trends were observed (Fig. S5).

**Table 3:** Pooled within-groups correlations between discriminating variables and standardized canonical discriminant functions.

	Function 1	Function 2
**% Variance**	64.4	35.6
**S1**	−0.7	0.01
**T23**	0.15	0.03
Mean mean frequency*	−0.12	−0.05
T12*	−0.06	0.01
Variance AM*	0.02	−0.01
**S3**	0.27	0.65
**S2**	0.34	−0.45
**Duration**	0.15	0.30
**Mean AM**	−0.04	0.21
**Variance Entropy**	0.14	−0.17
Variance mean frequency*	0.1	0.16
Variance FM*	−0.02	0.14
Mean FM*	0.05	0.13
Mean entropy*	−0.02	−0.06

Variables are ordered by absolute size of correlation within function. *: This variable was not used in the analysis.

### Intra-Individual Variability

#### Similarity between the two motifs produced by a given individual

As previously mentioned, European males and some hybrids produce two different motifs in a same bout. To describe the extent to which the sounds of the first motif match those of the second one, I used an automated method to measure similarity (see Material and Methods and [49] for further details). I observed a significant difference between the groups in the similarity (Kruskall-Wallis, H_5,67_ = 47.28, p<0.001, Fig. 3B and Fig. S1). The two motifs were structurally more similar in hybrids of the first generation (H1 and H2) than in EUR and backcrosses (BC1 and BC2). Therefore, backcrossing restored the ability to produce two motor programs that are very different, typical of the European quail to which the hybrid was backcrossed.

#### Changes of motif patterns within the breeding season

1/Intra-motif variability: the repetition of the same motif could exhibit within-bout variations. These variations did affect the spectral features of the syllables but not the syntactical structure of the crow (Fig. S6).

**Figure 5:**
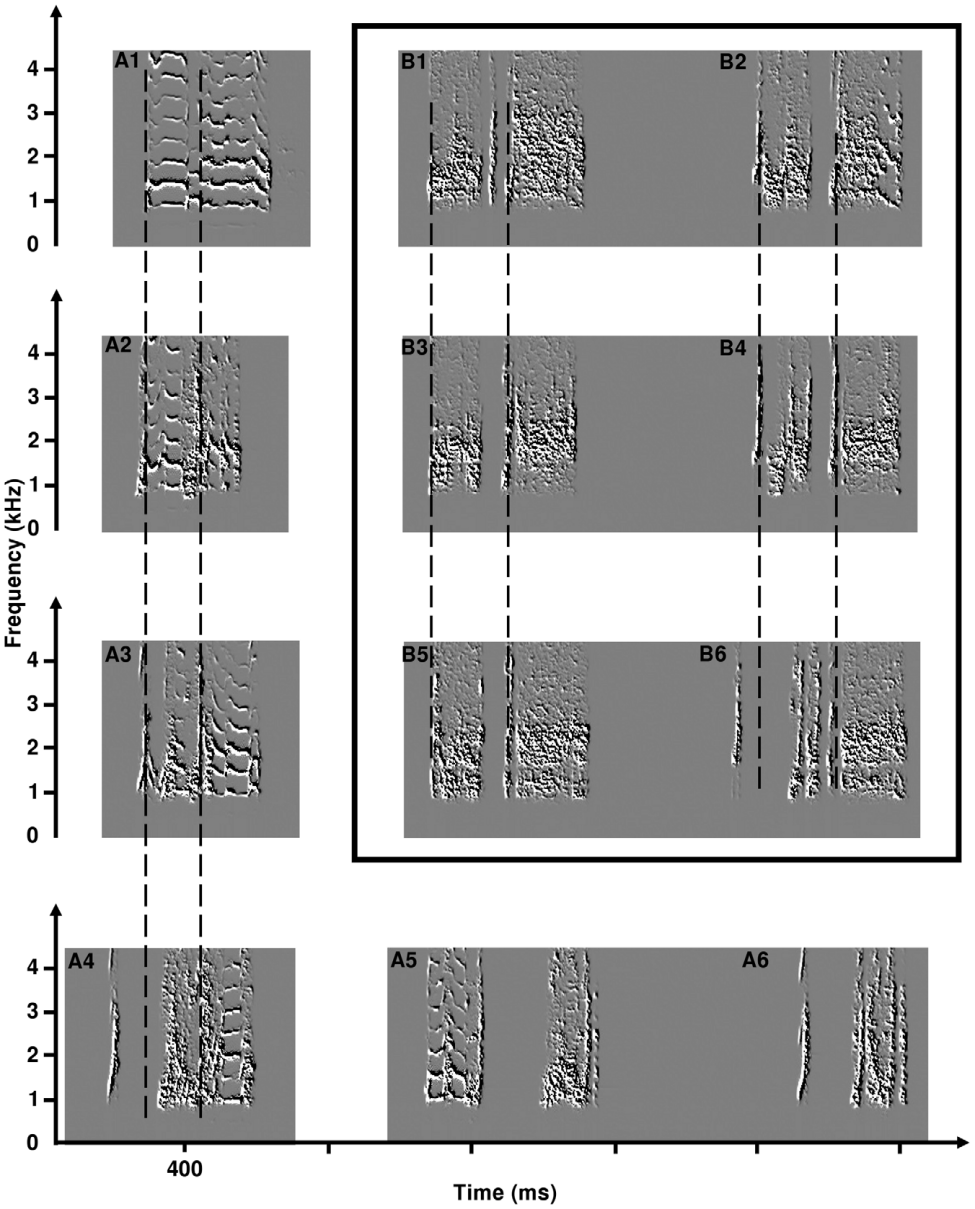
Spectrograms of hybrid crows, illustrating the intra-individual variability. A1 to A6: developmental stages of crows produce by one quail. A1: crow with a structure similar to the wawa produced by the European quail. A2 to A4: crows produced later during the recording session. A5 and A6: the two stable forms produced by the quail few days later. B1 to B6: developmental stages of crows produced by another quail. This quail always produced bouts with 2 successive crows. B1 and B2: crows produced at the beginning of the recording session. Note the similarities between the two motifs. B3 and B4: intermediary stage. Note the emerging differences between the 2 crows, and the differences between B2 and B4 (second crow of the bout). B5 and B6: stable motifs produced by this quail, produced about 30 minutes after the recording session started.

2/Changes of motif structure during ontogeny: In a few cases, I observed that the quail started to produce a sequence of motifs whose structures resembled those of the wawa of the European quail. This ‘original’ motif then exhibited some modifications and it was possible to record different unstable forms before the bird started to produce its stable motifs (Fig. 5, A1 to A6, A5 and A6 are the two stable motifs produced by this quail). Such vocal plasticity could be observed in hybrids that produce two motifs per bout. An example is illustrated on Figure 5 (B1 to B6). At the beginning of the recording session, this male started to produce two exemplars of the same motif per sequence (Fig. 5, B1 and B2). Then, the structure of the second motif of the sequence started to change gradually. About 30 minutes later, the male was producing two different motifs in the same sequence (Fig. 5, B5 and B6, these two motifs are the stable forms produced by this quail). Such changes in motif structure were observed during the breeding season, and were sometimes produced during the same recording session.

**Figure 6:**
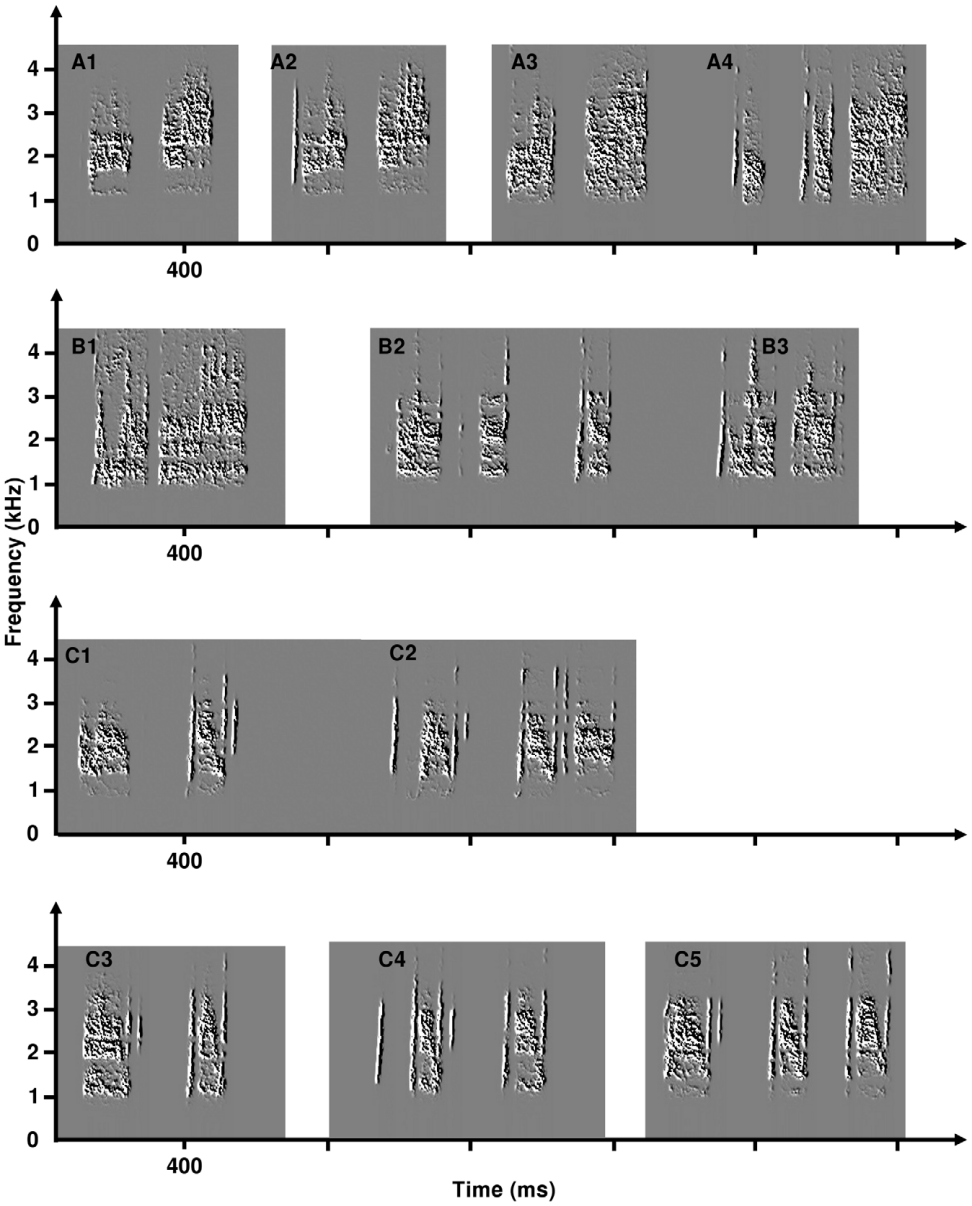
Spectrograms of hybrid crows, illustrating the intra-individual variability observed from one year to the other. A1 and A2: crows produced by one quail the first year. A3 and A4: crows produced by the same quail the second year. A1 and A3 are structurally similar; A4 constitutes a new syntactical form. B1 to B3: crows produced by a second quail. B1: single motif produced by this quail the first year. B2 and B3: crows produced by this quail during the second year. B1 and B3 are structurally similar. C1 to C5: crows produced by a third quail. C1 and C2: crows produced during the first year. C3 to C5: crows produced by the same quail the second year. C3 is structurally similar to C1; this is also the case for C2 and C5 despite that the intersyllabic gaps changed dramatically from one year to the other. Structure of the crow C4 is intermediary between C3 and C5. This accidental form occurred only once during the recording session, and could constitute an ‘accidental’ form.

3/Accidental motifs: like in European quail which produce sometimes some intermediary motifs between the wawa and the triplet, I observed during the same recording session the production of accidental motifs whose structure is intermediary between the two stable motifs produced by the individual (Fig. 6, motif C4). This could be the result of interference between the two different motor programs.

#### Changes of motif patterns between breeding seasons

Eight males H1 out of the 11 recorded in their second spring produced motifs whose structure resembled those observed in their first year. For the three remaining males, I observed some differences between years in the syntactical organization of the motif and/or in the spectral envelope of the signal (Fig. 6).

## Discussion

There are two main findings in this study. First, confirming previous studies on the acoustic signals of hybrids in different animal groups [5]–[13], [29], some hybrids crows were closer to one parental form but most of them presented a mosaic of characteristics of both subspecies. This study is original since hybrid crows were intermediate between the three types of crows produced by the two parental subspecies (since the European quail produces two different crows) and not between two parental signals as usually observed. Second, I observed in few cases some intra-individual variability in crowing patterns during a same recording session and across seasons, adding to the growing body of evidence that vocalizations produced by avian non learners are less stereotyped than previously thought [45], [51], [52].

Temporal structure is one of the most distinctive features differing between the crows produced by the two subspecies and their hybrids. Temporal parameters are relatively constant within individuals [44], [45] and are likely to be more reliable both for individual and species recognition [53]–[55]. This may suggest that selection had operated on temporal parameters in particular [56]. Spectral components of the crows might also be salient in species recognition. Contrary to a previous study [35], I did not find evidence that inheritance of tonal quality appear to be sex-linked. Indeed, hybrids of first generation H1 and H2 were not distinguishable based on spectral (and also temporal) features of their crows. Hybrid crows could be useful tools in playback experiments to investigate which acoustic features are important for species identification. It would be particularly interesting to check if there is a coupling between production and perception, i.e. if hybrids respond more strongly to the crow of the parental species they resemble most in their signals [57].

Like previously observed in doves [8], [27], backcrosses produced crows similar to the parental subspecies (European quail) to which they were backcross. It is likely that a similar result would have been obtained with backcrosses symmetric to BC1 and BC2 (i.e. male H1× female *japonica*; female H1× male *japonica*). So far, there has been no attempt to directly study the number of genes affecting crow production in the quail. The mixing of genes that occurs during hybridization might create new combinations of genes (from gene combinations that have already been shaped by evolutionary pressure in the parental subspecies), allowing new patterns to arise [30]. In addition, several studies have suggested that a number of genes that remain silent in modern birds can be reactivated upon appropriate signaling [58], [59] and it might be the case when two genomes that have been separated by thousands or million years met again following hybridization [60], [61]. Vocal production is a combination of different gestures (breathing, control of the vocal organ, head movements) [25], [62] and hybridization might give birth to unusual combinations [4].

Together with the high inter-individual variability of hybrid motifs, I also observed seasonal variations in crowing patterns, sometimes during a single recording session. This intra-individual variability might be due to ontogenetic changes recently described in the Japanese quail. In this study [45], crowing activity was continuously recorded in young males maintained in social isolation until sexual maturity. We observed developmental changes in crow structure, both the temporal and the spectral levels [45]. At the temporal level, three mechanisms can be observed: silence insertion, time warping in segment duration and time warping in inter-segment temporal intervals [45]. For example, in the Japanese quail, the first crow produced in life is often composed of two distinct segments. Silence insertion in the first or second segment gives rise to the characteristic tri-segmented structure of the Japanese quail's crow (Fig. 1) [45]. It is noteworthy that syntactical organizations observed during the development of the Japanese quail's crow constitute the stable forms produced by some hybrid quails (S0S and SS0S) [45], [63]–[64].

The intra-individual variability in hybrid motifs might exhibit a resilience of neotenic characteristics. It would be interesting to examine more carefully the stability of vocal signals produced by the two parental subspecies to validate this hypothesis. It might also be linked to testosterone production [65]. Testosterone treatment can induce crowing in hatchlings of Galliforms and gradual vocal changes have been reported [63], [64]. Quails with low levels of testosterone might have produced these unstable forms. I observed that 3 H1 males out of 11 exhibit vocal changes from one year to the other. Like in songbirds, these changes might be driven by changes in hormonal milieu, and the brain might experience a yearly rejuvenation [66].

Figure 2 presents a summary of the structural organization of hybrid motifs. It is based on both their inter- and their intra-individual variability. Some motifs resemble those produced by the two subspecies (Fig 2B: trisyllabic wawa of European quail; Fig. 2F: ‘classical’ wawa produced by the European quail; Fig. 2K: Japanese crow; Fig. 2O: triplet of the European quail). The remaining ones represent intermediaries that are not produced by sexually mature males of both subspecies. Nevertheless, some of these motifs resemble those observed during crowing ontogeny in both subspecies (Fig 2C and D) [45], [63]–[64]. Arrows indicate transitions between the different types of motifs. I observed such transitions between motifs during recording sessions (Fig. 5 and Fig. 6). These transitions could be described using mechanisms observed during crowing ontogeny in quails, namely silence insertion (SI) and modification of the spectral components (MSC) of the crows [45]. Syllable deletion (SD) is another mechanism that shape transition between two types of motif. Syllable deletions are observed in Japanese quails; the second syllable is sometimes accidentally omitted during vocal production (Derégnaucourt, *pers. obs*.).

Hybrid crows might represent an atavism (reappearance of an ancestral trait), but might also help to understand the route by which the parental species were formed in nature [4]. This schematic representation of hybrid crows' organization might also be used as a ‘tree of evolution’ to propose a possible scenario of vocal divergence that occurred during speciation. Inspired by the controversial Haeckel's theory [67]–[68], usually summarized as ‘ontogeny recapitulates phylogeny’ (1866), I propose that mechanisms observed during crowing ontogeny in quails might have been used to facilitate signal divergence during the speciation process. Starting from an ancestor signal, maybe a trisyllabic wawa (Fig. 2B), which is still produced by some males of the European quail, intermediary forms of quails might have transiently ‘crystallized’ some of these hybrid motifs (Fig. 2). Under different constraints, some of these crowing patterns might have disappeared or gradually evolved into the current patterns produced by the Palearctic quails. The two subspecies are morphologically similar and share the same habitat, so these ecological factors do not seem to have been involved. Nevertheless, transmission properties of the three crows produced by the two parental subspecies differ significantly. In the European quail, triplets are long-range signals whereas wawa serves predominantly in short-range interactions [39]. Japanese quail males tend to modulate amplitude depending on the social context [69]. It is more likely that signal divergence has been driven by sexual selection through male-male competition and female choice. Despite the fact that quails are not territorial [33], males of both subspecies responded more to conspecific than heterospecific crows and the response strength to hybrid signals was intermediate [48]. Intersexual use of crows has been described in the two subspecies using different experimental procedures [38], [41]–[43]. Species and mate-quality recognition are not independent of one another. The two processes might reinforce each other by jointly facilitating the speciation and diversification of sexually selected traits among closely related taxonomic groups [70]. As suggested above, it is likely that selection had operated on temporal parameters of the crow [56]. Females of the Japanese quail stimulated by a white noise following the specific rhythm developed faster sexually than a group in silence [42]. Female preference for a particular rhythm and vocal divergence of male signals during speciation might have undergone coordinated evolution as suggested for other animal models [71]. In a playback experiment of European, Japanese and hybrid crows, we observed that female European quails emitted more calls in response to the conspecific crows, an intermediary response in hybrid crows and less interest in the Japanese ones. Females of Japanese quail were not selective; we interpreted this as a result of domestication [38]. This result could also be explained by the sensory exploitation hypothesis, predicting that the evolution of sexually selected traits is influenced by some pre-existing sensory biases [72]. Several studies have also evidenced females' preferences for heterospecific signals, which were interpreted as ancestral traits [73]. Females of Japanese quail might have been sensitive to the wawa produced by the European quail, which according to the proposed scenario, could resembles the ancestral form produced before vocal divergence in Palearctic quails (see above). Additional experiments are required to validate this hypothesis.

## Materials and Methods

### 1. Experimental Subjects

I used domesticated Japanese quails from laboratory strains. As already described in the rooster [47], it is likely that domestication of the Japanese quail had little effect on crow structure [35], [74].

European quails have been bred in the laboratory of Rennes since the beginning of the 1980s, from wild birds caught in France, Spain and Portugal.

We produced hybrids from different combinations [37]: (1) female *japonica *× male *coturni*x: H1 (n = 15 breeding pairs); (2) female *coturnix *× male *japonica*: H2 (n = 8 breeding pairs); (3) female H1× male H1: F2 (n = 12 breeding pairs); (4) female H1× male *coturnix*: backcross 1: BC1 (n = 8 breeding pairs); (5) female *coturnix *× male H1: backcross 2: BC2 (n = 7 breeding pairs). This study forms parts of a European quail conservation program. Each year, tens of thousands of domestic Japanese quails are released on the reproductive areas of the European quail mainly in France, Italy, Spain and Portugal. The aim of this research program was to evaluate the risks of hybridization between the native European quail and the introduced Japanese quail [36]–[38], [75]. For time and logistic reasons, an emphasis was done on crossings that were more likely to occur in the field. This explains why hybrids of additional crossings (such as backcross with H2 or Japanese quail) were not produced.

### 2. Sound Recording and Analysis

The birds were reared in the laboratory aviary under the natural local photoperiod for Rennes (48°LN), and supplied with water and food (vitamin-supplemented pellets and wheat) *ad libitum*. They hatched in the summer and spent the autumn and the winter in unisex terrariums. At the beginning of their 1^st^ spring, males were transferred to individual cages, and their migratory impulse was recorded using infra-red detectors [75]. Their crows were recorded when they were completely sexually mature, as evidenced by the development of the proctodeal gland with foam production [76].

To investigate seasonal changes in the crow structure of hybrid males, I recorded 15H1 males several times during the breeding season. In addition, 11 male H1's were kept during the following winter in unisex terrariums. We checked their autumnal molt and their sexual regression, which were accompanied for some of them by a fat accumulation characteristic of migrating quails [75]. At the beginning of their 2^nd^ spring, they were transferred to individual cages and recorded as previously described.

Crows of individual males placed in a sound proof chamber were recorded with a Marantz CP 430 tape recorder equipped with a dynamic Sennheiser MD 41 microphone. During each recording session, I recorded about 10–30 crows from each individual.

#### Sound analysis

Crows were digitized using Goldwave (Goldwave Inc., version 5) sound recorder software at a frequency of 44100 Hz and at an accuracy of 16 bits. They were then analyzed using Sound Analysis Pro (SAP) version 2 [[49], [77], http://ofer.sci.ccny.cuny.edu/sound_analysis_pro] and results were stored in mySQL 4.0 tables (http://mySQL.com). Subsequent analysis was based on acoustic features computed on each spectral frame (10ms-window with 90% overlap): amplitude, pitch, Wiener entropy, and FM [49].

Number of motifs per bird: male European quails can emit some crows whose structure is intermediary between the wawa and the triplet sometimes (Guyomarc'h & Derégnaucourt, *pers. obs*.). These abnormal crows might be due to interference in the motor programs of these two vocalizations, and could be due for example to variations in motivation to crow. Such interferences have also been described in the coos emitted by doves [78]. Therefore, one could consider that hybrids produce two different motifs when two crows of different structure are produced in the same bout.

For the analysis of acoustic parameters, I considered only the stable motifs produced by hybrid males. Therefore, the number of motifs analyzed for each group is the following: 57 H1, 43 H2, 22 F2, 19 BC1 and 23 BC2.

Measures of acoustic parameters of crows: at the temporal level, I measured the duration of the different segments that constitute a crow (Fig. 1). Duration of segments was delineated by SAP (thresholds: amplitude >25dB, entropy <−2.2). In both subspecies and hybrids, the temporal pattern of the crow is marked by sharp transitions in amplitude thresholds. In the wawa of the European quail, such a transition is observed around the middle of the crow (wa-wa). The triplet of the European quail is well known for its brief, clear loud 3 notes (2 notes composed the second part of the crow). Such a 3-pulse rhythm is observed in the crow of most Japanese quails, which is composed of three parts separated by silence. The temporal pattern of the Japanese quail's crow is different from the triplet: the interval between the first note and the second note of the triplet is significantly longer than the interval between the first part and the second part of the Japanese quail's crow (Fig. 1).

Regarding the spectral envelope, the wawa of the European quail sounds like a grunt, and is composed of a low-pitch vibrato [39]. The triplet is composed of warbled elements with vibrato. The Japanese quail's crow is composed of noisy parts, with a fast trill in the final part of the signal. Since the two subspecies produce different types of sounds, and hybrids often combined them, it was not relevant to use spectral parameters that were used in previous studies at the subspecific level (e.g. minimum frequency of the triplet's pulses; [44]). Therefore, acoustic parameters were computed over the complete crow (i.e. one single value for each acoustic feature per crow). Different spectral parameters were taken into account (mean and variance for each parameter, [49]): 1/Frequency modulation: an estimate of the absolute slope of frequency traces; 2/Wiener Entropy: entropy measures the width and uniformity of a power spectrum on a logarithmic scale: white noise corresponds to 0, a pure tone to minus one; 3/Mean frequency (Hz): a smooth estimate of the center of derivative power; 4/Amplitude modulation: changes in the amplitude envelope per unit of time.

For hybrids that produced two different motifs in a same bout, similarity between the two signals was calculated using SAP [49]. It is the product of two measures: similarity score and accuracy score. These measures were obtained from asymmetric pairwise comparisons. In asymmetric comparisons, the most similar sound elements of two motifs are compared, independent of their position within a motif. The smallest unit of comparison is a 10ms-long sound interval (FFT windows). Each interval is characterized by measures for four acoustic features: pitch, FM, amplitude modulation (AM) and Wiener entropy. SAP calculates the Euclidean distance between all interval pairs from two motifs, over the course of the motif, and determines a p-value for each interval pair. The percentage of overall significant similarity between the two motifs represents the similarity score; it thus reflects how much of similar sound material was found in both motifs. To measure how accurate are the sound elements similar in both motifs at a fine scale level, I also used the accuracy score from SAP. This score is computed locally, across short (10 ms) FFT windows and indicates how well the sound matched in both motifs. SAP calculates an average accuracy value of the motif by averaging all accuracy values across the similarity segments.

### 3. Statistical Analysis

Kruskal-Wallis and Mann-Whitney tests were used to test differences between groups and the Chi-square test was used to compare frequencies between the different groups [79].

Using a discriminant function analysis (DFA), I tested whether hybrid crows were more similar to those of the European or the Japanese quail. I constructed a discriminant function analysis from all acoustic variables from the three calls produced by the two parental subspecies. The analysis (F to enter: 3.84, F to remove: 2.71) resulted in one discriminant function that classified individuals according to the three different types of calls. The variables used for the analysis were individual means of acoustic parameters. Jackknife cross-validations were applied to the classification procedures. All analyses were done with SPSS version 15.

## Supporting Information

Figure S1 Spectrograms of crows produced by hybrid quails. Like European quails, some hybrid quails can produce two different motifs in a same bout. Each letter represents one individual. Similarity score (%) between the two motifs, calculated by Sound Analysis Pro, is indicated for each individual.
(2.26 MB TIF)

Figure S2 Temporal components of crows produced by Japanese quails, European quails and their hybrids. Median scores are represented by central lines, interquartile ranges by boxes, 10th and 90th percentiles by whiskers and extreme values by black squares. TD: Total Duration; S1, S2, S3: duration of Segment 1, 2, 3; T12: time interval between impulsion 1 and impulsion 2; T23: time interval between impulsion 2 and impulsion 3. H1: female *japonica *× male *coturnix*; H2: female *coturnix *× male *japonica*; F2: female H1× male H1: BC1: female H1× male *coturnix*; BC2: female *coturnix *× male H1; EUR1: wawa of the European quail; EUR2: triplet of the European quail; JAP: *japonica*.
(0.25 MB TIF)

Figure S3 Spectral components of crows produced by Japanese quails, European quails and their hybrids. Median scores are represented by central lines, interquartile ranges by boxes, 10th and 90th percentiles by whiskers and extreme values by black squares. H1: female *japonica *× male *coturnix*; H2: female *coturnix *× male *japonica*; F2: female H1× male H1; BC1: female H1× male *coturnix*; BC2: female *coturnix *× male H1; EUR1: wawa of the European quail; EUR2: triplet of the European quail; JAP: *japonica*.
(0.38 MB TIF)

Figure S4 Results of the classification following the discriminant function analyses, taken into account all acoustic parameters. A: hybrid crows not assigned to a separate group. B: hybrid crows assigned to a separate group. H1: female *japonica *× male *coturnix*; H2: female *coturnix *× male *japonica*; F2: female H1× male H1; BC1: female H × male *coturnix*; BC2: female *coturnix *× male H1; EUR1: wawa of the European quail; EUR2: triplet of the European quail; JAP: *japonica*.
(0.29 MB TIF)

Figure S5 Results of the classification following discriminant function analyses, taking into account only the spectral components of the crows. A: hybrid crows not assigned to a separate group. B: hybrid crows assigned to a separate group. H1: female *japonica *× male *coturnix*; H2: female *coturnix *× male *japonica*; F2: female H1 × male H1; BC1: female H1 × male *coturnix*; BC2: female *coturnix *× male H1; EUR1: wawa of the European quail; EUR2: triplet of the European quail; JAP: *japonica*.
(0.23 MB TIF)

Figure S6 Spectrograms of crows produced by hybrid quails, illustrating the intra-individual variability. A1 to A5: one quail started to produce sequences composed of repetitions of a single syllable (A1 to A3). One can observe a gradual occurrence of a note at the beginning of the syllable. A4 and A5: later on, this quail produced bouts composed of two motifs that slightly differ in their spectral envelope. B1 and B2: crows produced by another quail, in a same bout. Note the similarities between the two crows, despite the differences in the spectral envelope of the first and the second syllable.
(0.94 MB TIF)
