# Supplementary figures and images for: Interspecific Hybridization as a Tool to Understand Vocal Divergence: The Example of Crowing in Quail (Genus Coturnix)
Source: PLoS One. 2010 Feb 26;5(2):e9451. doi: 10.1371/journal.pone.0009451 (PMC2829089; doi:10.1371/journal.pone.0009451)

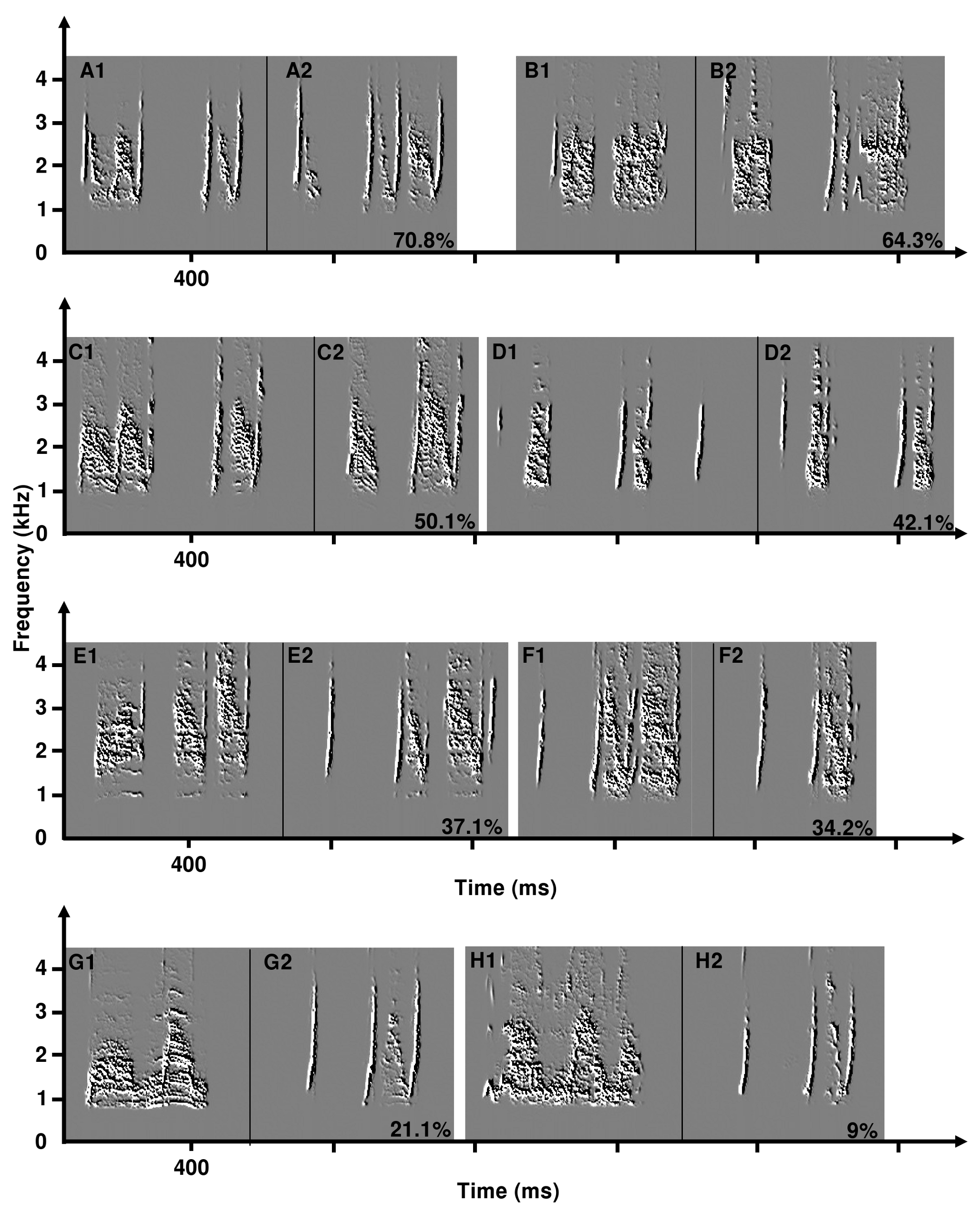

Supplement: Figure S1 — Spectrograms of crows produced by hybrid quails. Like European quails, some hybrid quails can produce two different motifs in a same bout. Each letter represents one individual. Similarity score (%) between the two motifs, calculated by Sound Analysis Pro, is indicated for each individual. (2.26 MB TIF) [file pone.0009451.s001.tif]

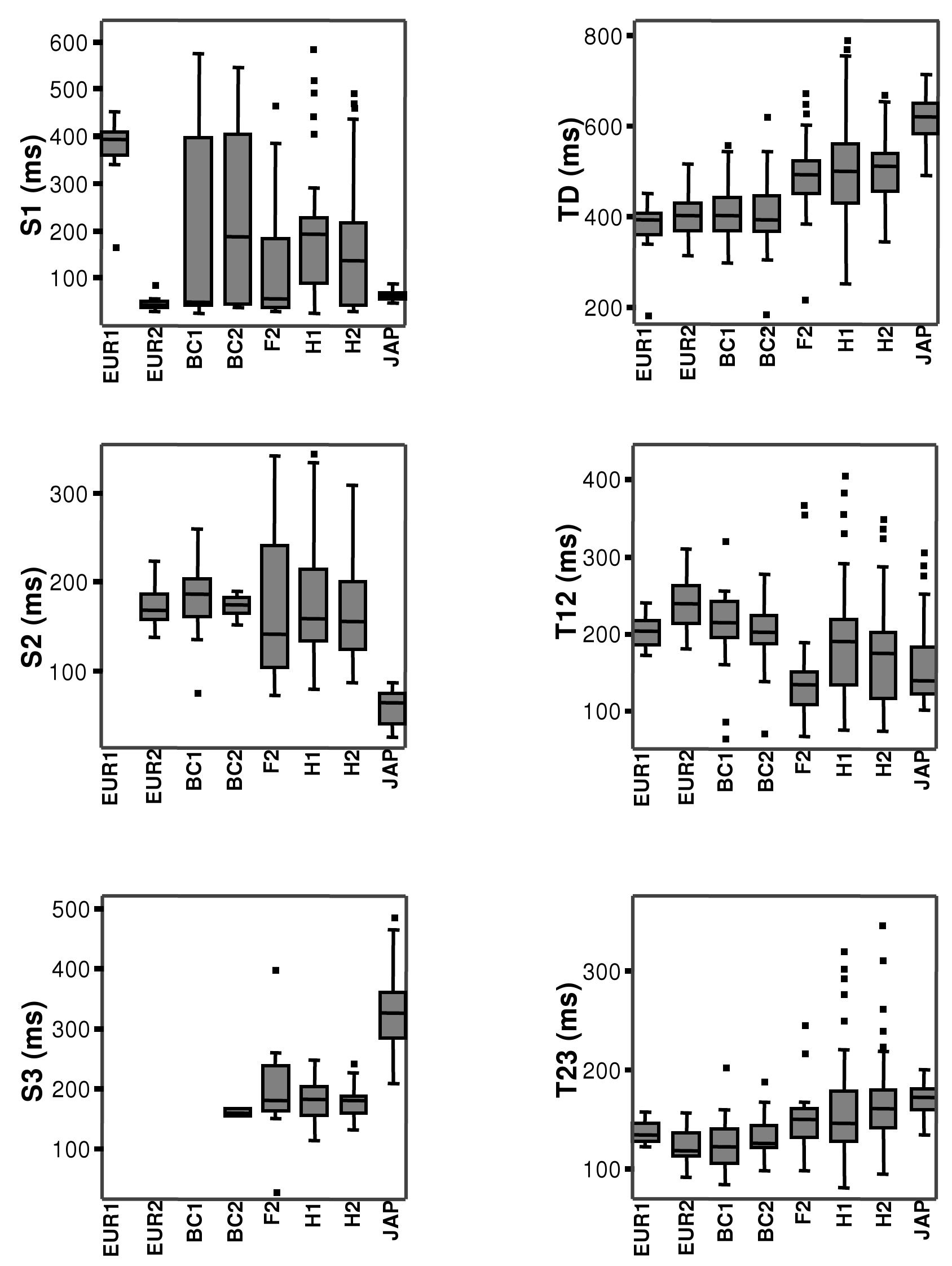

Supplement: Figure S2 — Temporal components of crows produced by Japanese quails, European quails and their hybrids. Median scores are represented by central lines, interquartile ranges by boxes, 10th and 90th percentiles by whiskers and extreme values by black squares. TD: Total Duration; S1, S2, S3: duration of Segment 1, 2, 3; T12: time interval between impulsion 1 and impulsion 2; T23: time interval between impulsion 2 and impulsion 3. H1: female japonica × male coturnix; H2: female coturnix × male japonica; F2: female H1× male H1: BC1: female H1× male coturnix; BC2: female coturnix × male H1; EUR1: wawa of the European quail; EUR2: triplet of the European quail; JAP: japonica. (0.25 MB TIF) [file pone.0009451.s002.tif]

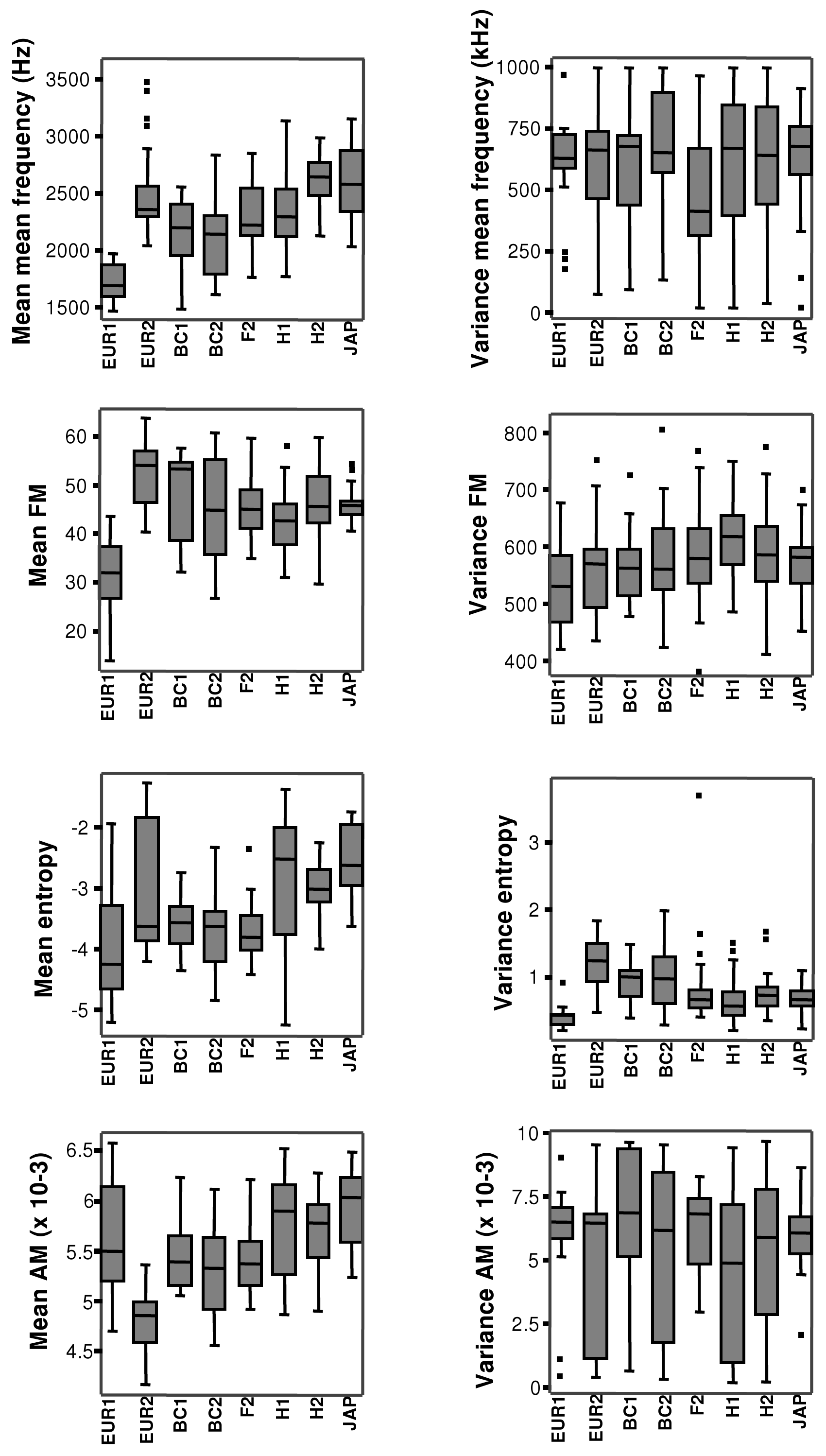

Supplement: Figure S3 — Spectral components of crows produced by Japanese quails, European quails and their hybrids. Median scores are represented by central lines, interquartile ranges by boxes, 10th and 90th percentiles by whiskers and extreme values by black squares. H1: female japonica × male coturnix; H2: female coturnix × male japonica; F2: female H1× male H1; BC1: female H1× male coturnix; BC2: female coturnix × male H1; EUR1: wawa of the European quail; EUR2: triplet of the European quail; JAP: japonica. (0.38 MB TIF) [file pone.0009451.s003.tif]

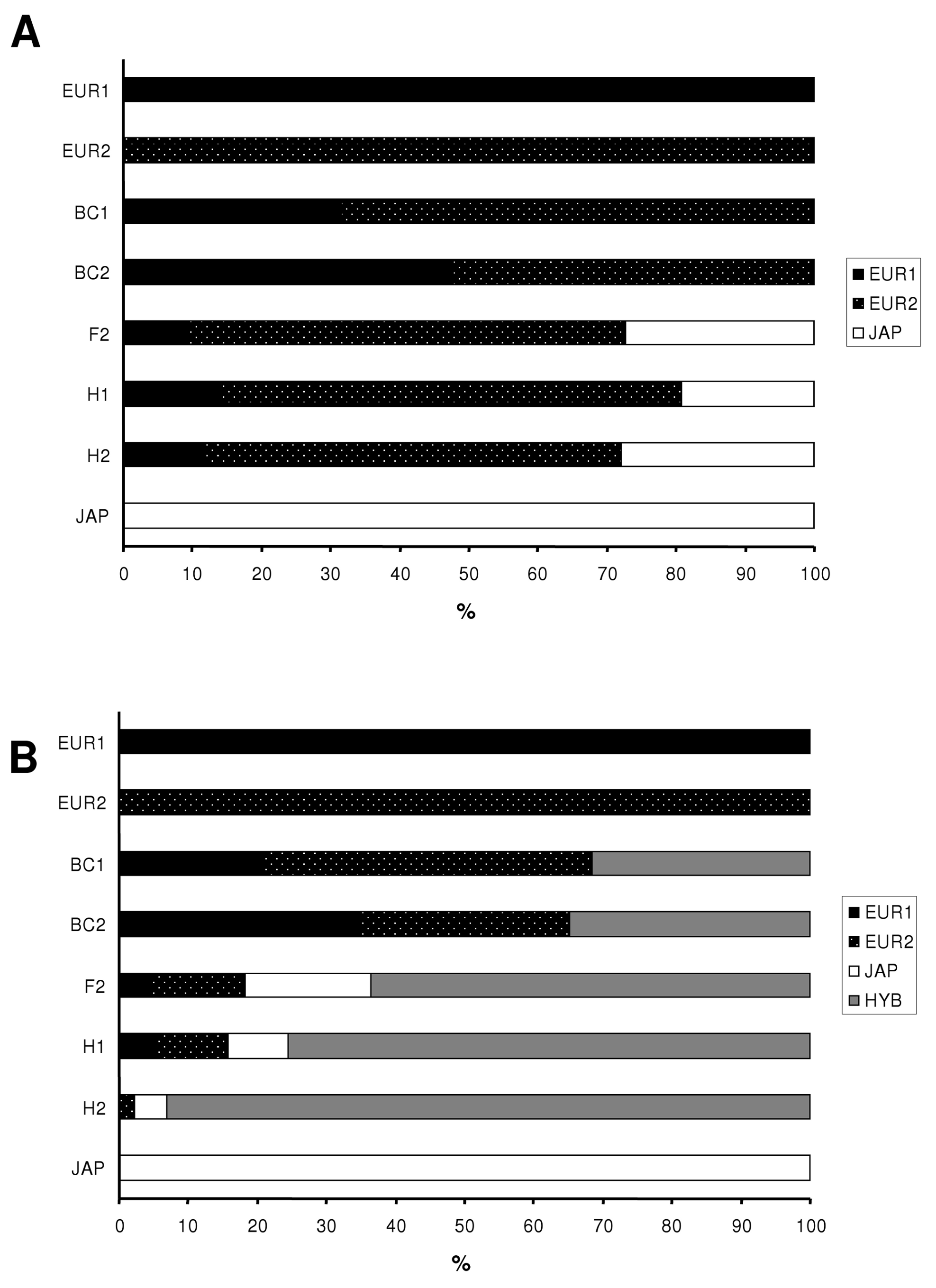

Supplement: Figure S4 — Results of the classification following the discriminant function analyses, taken into account all acoustic parameters. A: hybrid crows not assigned to a separate group. B: hybrid crows assigned to a separate group. H1: female japonica × male coturnix; H2: female coturnix × male japonica; F2: female H1× male H1; BC1: female H × male coturnix; BC2: female coturnix × male H1; EUR1: wawa of the European quail; EUR2: triplet of the European quail; JAP: japonica. (0.29 MB TIF) [file pone.0009451.s004.tif]

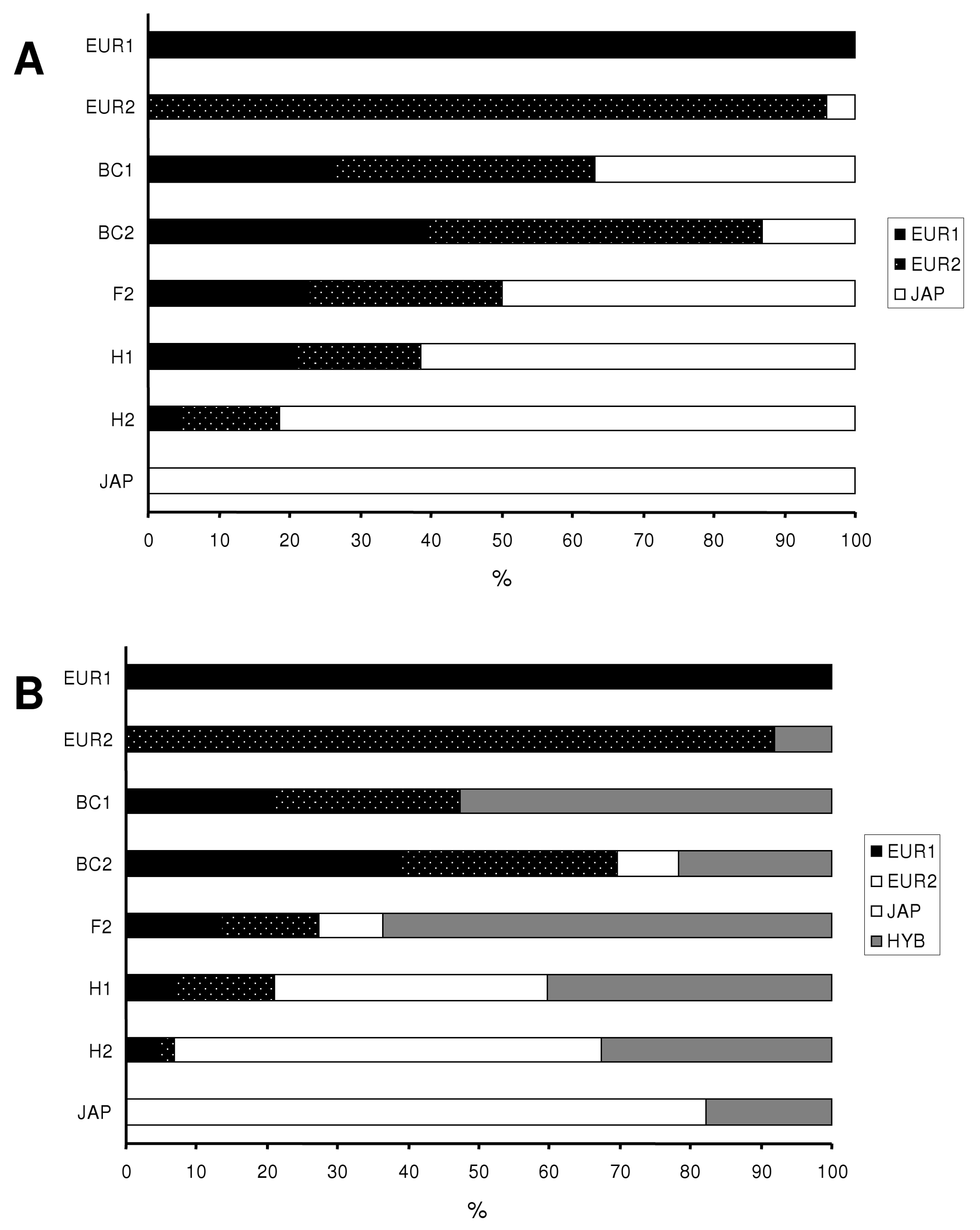

Supplement: Figure S5 — Results of the classification following discriminant function analyses, taking into account only the spectral components of the crows. A: hybrid crows not assigned to a separate group. B: hybrid crows assigned to a separate group. H1: female japonica × male coturnix; H2: female coturnix × male japonica; F2: female H1 × male H1; BC1: female H1 × male coturnix; BC2: female coturnix × male H1; EUR1: wawa of the European quail; EUR2: triplet of the European quail; JAP: japonica. (0.23 MB TIF) [file pone.0009451.s005.tif]

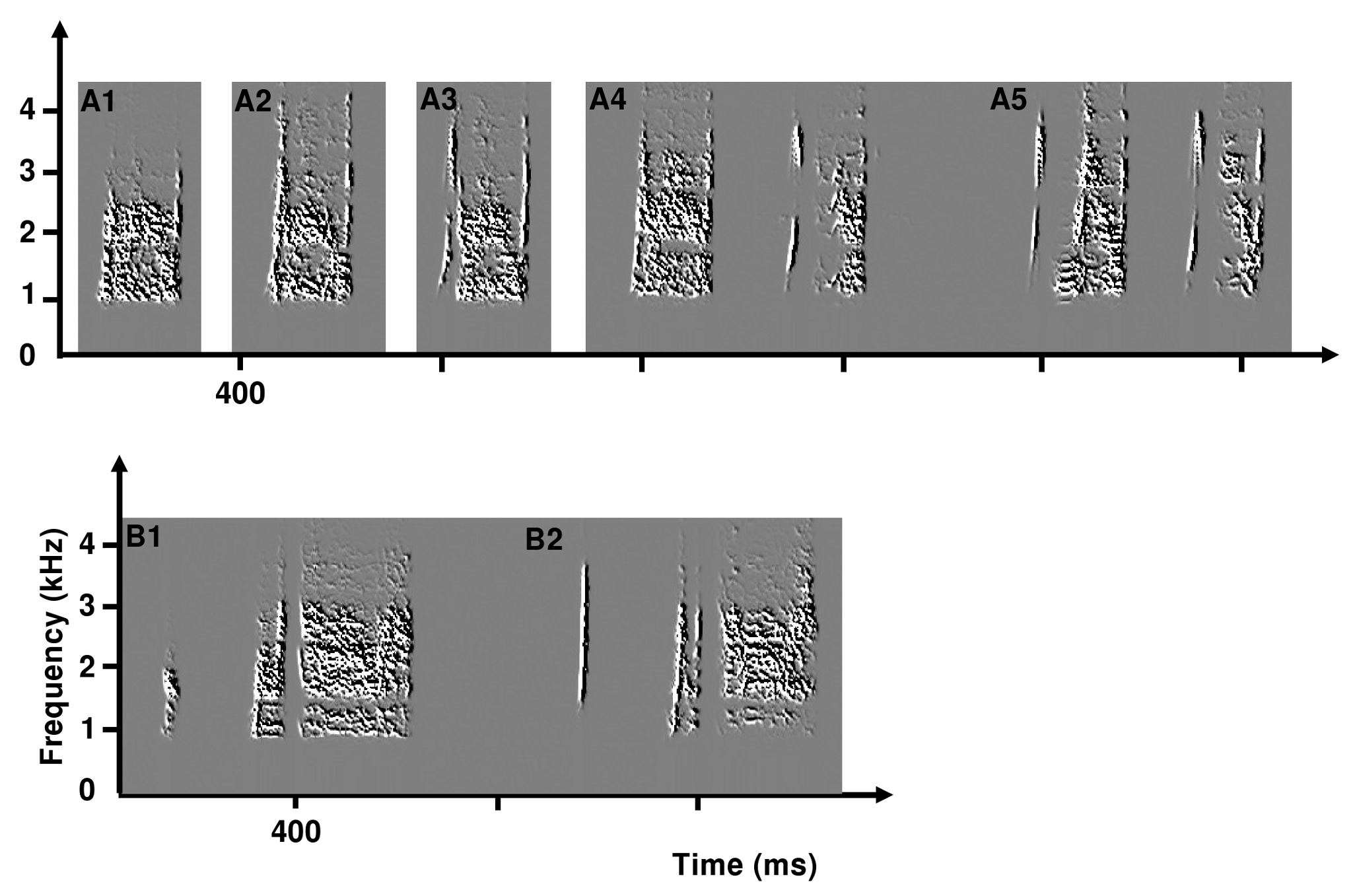

Supplement: Figure S6 — Spectrograms of crows produced by hybrid quails, illustrating the intra-individual variability. A1 to A5: one quail started to produce sequences composed of repetitions of a single syllable (A1 to A3). One can observe a gradual occurrence of a note at the beginning of the syllable. A4 and A5: later on, this quail produced bouts composed of two motifs that slightly differ in their spectral envelope. B1 and B2: crows produced by another quail, in a same bout. Note the similarities between the two crows, despite the differences in the spectral envelope of the first and the second syllable. (0.94 MB TIF) [file pone.0009451.s006.tif]
